# Re-engineering contested concepts. A reflective-equilibrium approach

**DOI:** 10.1007/s11229-022-03556-7

**Published:** 2022-04-15

**Authors:** Georg Brun

**Affiliations:** Institute for Philosophy, University of Bern, Länggassstrasse 49a, 3012 Bern, Switzerland

**Keywords:** Conception, Conceptual engineering, Contested concepts, Explication, Rawls, Reflective equilibrium

## Abstract

Social scientists, political scientists and philosophers debate key concepts such as *democracy, power* and *autonomy*. Contested concepts like these pose questions: Are terms such as “democracy” hopelessly ambiguous? How can two theorists defend alternative accounts of democracy without talking past each other? How can we understand debates in which theorists disagree about what democracy is? This paper first discusses the popular strategy to answer these questions by appealing to Rawls’s distinction between concepts and conceptions. According to this approach, defenders of rival conceptions of, e.g. justice can disagree without talking past each other because they share the concept of justice. It is argued that this idea is attractive but limited in application and that it fails to do justice to the dynamic and normative aspects of concept formation. Reflective equilibrium is then suggested as an alternative approach. It replaces the static contrast between a conceptual ‘core’ and competing conceptions by a dynamic perspective of concept formation as a partly normative undertaking: pre-theoretic language use and commitments can provide a shared starting point for developing alternative accounts which yield different concepts of, e.g. justice. This perspective provides a new understanding of how it is possible that different theorists defend rival accounts of, e.g. justice, without talking past each other.

## Introduction

Social scientists, political scientists and philosophers debate key concepts such as *autonomy, democracy, freedom, justice, power* and *race*.^1^ It may even seem that a great deal of the debates is just about how such contested concepts should best be understood. This poses questions: Are terms such as “democracy” hopelessly ambiguous? How can debating theorists defend alternative accounts of, for example, democracy without talking past each other? How can we understand debates in which theorists disagree about what democracy is? The principal aim of this paper is to investigate what the idea of a reflective equilibrium can contribute to answering such questions in a context of theory development in which contested concepts such as *democracy* play a key role.^2^ Of course, ambiguity and talking past each other can plague any dispute, but contested concepts appear to be an especially productive source of such problems. The very possibility of talking past each other is therefore hardly puzzling, but it is a challenge to explain how it is possible to disagree about contested concepts and defend rival theoretical accounts without talking past each other. As I will argue, a reflective-equilibrium approach can be used to give such an explanation.

The idea of analysing debates about contested concepts with the help of reflective-equilibrium theory has hardly ever been explored so far.^3^ I will therefore begin (in Sect. 2) by discussing another, popular, way of thinking about the problems raised by contested concepts, namely the idea of relying on Rawls’s distinction between concepts and conceptions (which is independent of his account of reflective equilibrium): different theorists can share the concept of, for example, democracy, but still debate different interpretations of it by proposing different conceptions of democracy; adherence to the conceptual ‘core’ ensures that using “democracy” does not lead them into talking past each other and that the proposed conceptions are conceptions *of democracy.* An analysis of what makes the concept-conception approach attractive and why it is, as I will argue, ultimately unsatisfactory will provide the backdrop for the subsequent discussion of how reflective equilibrium can deal with the challenge of theorizing in a context in which contested concepts play a key role. The aim of discussing Rawls’s concept-conception distinction is not to refute it, but to provide a point of reference for my own proposal, which I expect to avoid the limitations and challenges I diagnose for the concept-conception approach while retaining the features that make it attractive.

Section 3 turns to the main objective of the paper and introduces reflective equilibrium. I first sketch (in Sect. 3.1) the version of reflective-equilibrium theory I am relying on. Section 3.2 then explains how reflective equilibrium can be interpreted as including an account of how concepts are re-engineered in the course of theory development, specifically an account of explication as described by Carnap (1962). On this basis, I investigate how contested concepts can be dealt with by reflective equilibrium. In a first step (Sect. 4.1), I analyse whether and how the concept-conception distinction could be reconstructed in a reflective-equilibrium framework. The result is that cases of theory development which include conceptual re-engineering of, for example, *democracy* resist an interpretation in terms of the concept-conception distinction, because the static picture of the concept of democracy as a common ‘core’ for rival conceptions of democracy cannot be squared with the dynamics of theory development. As an alternative, Section 4.2 develops the idea that the common conceptual ‘core’ should be replaced by a shared starting point in pre-theoretic language use of, for example, “democratic” and shared commitments about democracy. Drawing on Carnap’s theory of explication, I suggest that if two theorists can make it practically clear that they share such a starting point, this can explain how it is possible that they can defend rival accounts without talking past each other. In this way, it is argued, we can take up those aspects of the concept-conception distinction that make it attractive in the first place. Finally, Section 5 explores how the suggested reflective-equilibrium perspective helps to understand theoretical disputes revolving around contested concepts. In particular, it briefly discusses the various ways in which it is possible, within a reflective-equilibrium framework, for two theorists to arrive at rival accounts without talking past each other.

## The concept-conception distinction

The concept-conception approach shares two peculiarities with many other influential methodological ideas in philosophy: it has its origins in brief remarks in classic works and it exists in many varieties, most of them not exactly defined.^4^ This is often a source of problems because methodological remarks in classic works tend to be short, sketchy yet insightful explanations in the introductory part, not necessarily fully in line with what the author subsequently does. Consequently, many different, usually again only sketched, varieties of a methodological idea circulate in the literature, all of them seen as representing the original idea or a further development thereof by their advocates.

For the concept-conception distinction, the most influential source is Rawls’s *A Theory of Justice*, where he explains that his conception of justice as fairness can be seen as an interpretation of the concept of justice which he thinks is common to different conceptions.^5^ Subsequently, the distinction has been applied to a wide range of concepts and many different characterizations of it have emerged.^6^ In what follows, I will not attempt to give an overview of the diversity of ideas that have been proposed under the label “concept vs. conception”.^7^ Instead, I focus on Rawls’s explicit remarks (1999:5, 9; see also 1980, 2005:14n15) with the goal of presenting a plausible understanding of his core idea.^8^

### Rawls’s classic account

Subsequent re-interpretations and variations notwithstanding, Rawls’s basic line of thought is clear enough. Defenders of different conceptions of, for example, justice are involved in a meaningful dispute; that is, they are not merely talking past each other, as they would if they associated different meanings with the term “justice”. So the challenge is to understand debates centred around contested concepts in a way that does not rest on an appeal to ambiguity or straightforward context-dependency.^9^ The concept-conception approach meets this challenge by assuming that concepts constitute a common ‘core’ of different conceptions. For example, Rawls characterizes the concept of justice as follows: “institutions are just when no arbitrary distinctions are made between persons in the assigning of basic rights and duties and when the rules determine a proper balance between competing claims to the advantages of social life.” (Rawls 1999:5) He then explains that this expresses that the concept of justice “includes” (1999:5) and is “defined” (1999:9) by a range of “notions” such as *arbitrary distinction* and *proper balance*, and that “interpreting” (1999:5) these component-notions is the task of a conception of justice, which has to “specify” or “define” (1999:5, 9) what counts as, for example, a proper balance or an arbitrary distinction. Spelling out a conception of justice is, in turn, a main task of a theory of justice. What goes on in debates that involve the contested concept of justice can be explained by pointing out that defenders of different conceptions can disagree on principles of justice and hence on theories of justice while still sharing the concept of justice. The shared concept settles only the relevant meaning of the term “justice”; a conception of justice is needed for interpreting the notions which define “justice”, and defenders of rival conceptions of justice disagree on how these notions should be interpreted, and therefore typically also on what actually counts as just. In Rawls’s analysis and terminology, the disagreement in a debate in which a contested concept plays a key role does not really concern the concept but rather the conceptions.^10^

That there is a one-to-many relation between concepts and conceptions does not imply that all conceptions are on a par. It makes perfect sense to evaluate conceptions in comparison with one another, either with respect to the concept (do they deliver a consistent and complete account of the component-notions?), or relative to some ‘external’ considerations such as the purpose the conception is meant to serve, background assumptions or empirical evidence. This draws attention to the important point that conceptions can answer different problems (Rawls 1980:571). Rawls (1999:6–9), for example, makes clear that he seeks to develop an ideal-theory account of justice applicable to the basic structure of society and carefully explains how exactly he understands this objective.

The concept-conception distinction is attractive because it promises a reconstruction of debates centred around contested concepts which explains how it is possible that defenders of alternative accounts disagree without talking past each other. Such an explanation seems plausible, for instance, for a debate about enfranchisement in which disputants readily agree on the concept of enfranchisement by accepting that “*x* is enfranchised” applies to exactly those who have been given voting rights in the relevant context (e.g. nation wide elections in a given state), but still defend different principles of enfranchisement which appeal to prospective in contrast to retrospective residency or to different competence requirements, different age-restrictions and so on (see, e.g. Beckman 2009:ch. 1).^11^

In addition, the concept-conception distinction may also seem to contribute something helpful to theory construction (although I did not find defenders suggesting this). If a concept can be identified, its definition can be used as a blueprint for constructing a conception by defining or interpreting the component-notions (e.g. Rawls’s concept of justice calls for defining which distinctions count as arbitrary).

However, one may have serious doubts whether the concept-conception distinction can reliably deliver on these promises, despite its plausibility when applied to certain specific cases like the debate about enfranchisement.

### Limitations and challenges

A first kind of worry is that the range of debates which can be adequately analysed with the help of the concept-conception distinction may be surprisingly limited. There are several factors that render many proposed applications of the distinction questionable. In some cases which are claimed to instantiate the concept-conception structure, the agreed ‘core’ actually determines a hyperonym of the concept in question.^12^ But agreeing on a hyperonym is not enough to explain why defenders of different conceptions that target different hyponyms do not talk past each other.^13^ They can easily talk past each other by unknowingly referring to different hyponyms. That they agree on an hyperonym will then even make it more difficult to spot the problem if the term used for the target concept covers both a hyperonym and (some of) its hyponyms (so-called vertical polysemy; see Lanius 2019:20), for example, if “justice” is used not only for Rawls’s justice as fairness, but also in the more general sense that covers justice as fairness as well as its (e.g. utilitarian) competitors. If, on the other hand, it is acknowledged that the common ‘core’ merely fixes a hyperonym (as for example Newman et al. 2017:27 do with respect to *intrinsic value*), then this ‘core’ concept is not the contested concept in focus and the concept-conception approach misdescribes a dispute between, for example, theorists who talk about direct democracy and theorists who talk about representational democracy as a dispute about *democracy*.

In other cases, disagreements which seem not to reduce to talking past each other resist an explanation by the concept-conception distinction since they go right to the conceptual ‘core’. This point was convincingly argued by Swanton (1992:viii–ix; 24) with reference to the disagreement about *freedom*, which involves irreconcilable disputes even over the formal structure of *freedom* when theorists debate whether “free” is a one-, two- or three-place predicate. Hence defenders of the concept-conception approach may ask for too much if they insist on one common concept with a clearly structured meaning. Less could suffice to ensure that debaters do not talk past each other, for example an agreement on paradigmatic instances and non-instances (in the case of *democracy*, e.g. undisputed instances of democratic and non-democratic societies such as Sweden and North Korea), some merely necessary or merely sufficient conditions (maybe that democracy needs some form of voting), or some ‘symptoms’ (e.g. in democratic societies voters typically have a choice between substantially different options).

More important than problems affecting specific applications of the concept-conception distinction is the principled worry that the concept-conception approach stands in the way of adequately accounting for the dynamic and normative aspects of concept formation which are characteristic in the context of theory development. It does not factor in that developing conceptions – especially philosophical, not merely everyday, conceptions – is a goal-directed and temporally extended activity, which often includes efforts in concept formation. The concept-conception approach therefore gives unsatisfactory results in cases in which dynamic or normative aspects of theory development play a vital role for understanding a dispute revolving around a contested concept.

The claim that the concept-conception approach does not do justice to the dynamics of theory development is not, of course, meant to imply that theorists who invoke the concept-conception distinction are not actively involved in theory development; quite the contrary is true. Nonetheless, the explicit descriptions of the concept-conception contrast draw a static picture of a concept as the ‘core’ of a number of conceptions; they do not refer to the understanding of a concept at different stages of theory development. The problem is not merely that the concept-conception contrast does not explicitly account for the dynamics of theory development, but rather that, once this dynamic aspect is taken seriously, a new perspective emerges which resists an easy integration into the concept-conception approach.

As the concept-conception approach understands the idea of a common ‘core’, concepts are “included in” (Rawls 2005:14n15) or shared by various conceptions, but from the dynamic perspective, we also have to answer the questions of whether the concept of *x* need be given at the outset of inquiry, before one or more conceptions of *x* are developed; and if a concept is needed at this start-of-inquiry stage, whether we also need a definition of it, or at least need to know what its component-notions are. Several considerations indicate negative answers. Before and independently of developing a conception, one may simply fail to have an idea of how the relevant concept could be defined. It may even be the case that the component-notions cannot be identified because resources of the target conception are needed to do this, for example, certain distinctions. Moreover, the process of developing a conception can uncover reasons to revise a concept, for example, if it discloses that a concept is actually inconsistent, ambiguous or excessively vague. A vivid illustration of all the points just mentioned can be found in Hardimon’s (2017) analysis of concepts and conceptions of race. His theoretical account develops a number of different concepts and conceptions, which in turn provide the very basis for uncovering conflations, ambiguities and inconsistencies in the customary use of the term “race”. Hardimon also proposes revisionary definitions and relies on notions that cannot be assumed to be available before an attempt at spelling out a conception is made; for example, notions from biological theory, which are needed to define a scientific version of *race* (called “populationist race” by Hardimon). All this shows that concepts need not exist at the start of inquiry, but can also be a result of developing a conception. In line with this diagnosis, it is sometimes argued that “carving out” a concept is one of the tasks a conception should accomplish (Seidel 2016:34). These points do not render the concept-conception approach useless, but they show three things.

First, the scope of application of the concept-conception approach would be very limited if it would be required that a concept is known, or even explicitly defined, before the conceptions which are supposed to include it as a common ‘core’ have been developed. Second, the practical advice I considered above may regularly prove unhelpful as a procedure for developing conceptions, because it will not or cannot be known beforehand what the component-notions are, which a conception is supposed to deal with. Third, the concept-conception distinction is best understood as applicable to the results of theory development, but not necessarily to earlier stages of theory development. This makes room for admitting that concepts are often a result of developing a conception without undermining Rawls’s central idea, namely: the fact that different conceptions include the same concept as a common ‘core’ explains why defenders of rival conceptions do no talk past each other.

However, once it is admitted that concepts are often a result of developing a conception, it becomes even more doubtful whether the concept-conception distinction can in fact be successfully applied to a significant number of the debates about contested concepts that have been presented as exhibiting a concept-conception structure. Of course, that a concept was formed as a result of developing a conception does not entail that it is not also part of other conceptions. But if somebody claims as a common ‘core’ a concept she has adopted as a result of developing a specific conception, she incurs a considerable burden of proof: she has to show that the alternative conceptions include this very same concept as well. This burden is rarely discharged.^14^ As long as this has not been done, it is hard to suppress the suspicion that the conception has left its marks on the allegedly common concept such that it is not acceptable to defenders of other conceptions. It is, for instance, a pretty obvious yet crucial move to ask whether other theorists are actually ready to accept Rawls’s concept of justice. And it is hardly surprising that some defenders of other conceptions were quick to reject Rawls’s concept (e.g. Flew 1985; Matson 1978).

The normative aspect poses similar challenges. Developing a conception is typically a normative undertaking in that it involves the proposal that a certain concept *should be adopted*. Of course, this is not how Rawls, for example, frames what he is doing when he introduces the concept of justice early on in *A Theory of Justice*, where he presents himself as giving a “description” of ‘the’ concept of justice (Rawls 1999:5). But as Plunkett and Sundell have recently insisted (e.g. Plunkett/Sundell 2013; Plunkett 2015), declarative utterances are routinely used – in everyday communication and in philosophy – normatively as proposing to use a term in a certain way. For example, one may say “North Korea is not a democracy” to convey that one holds that “democracy” should not be used in a sense that classifies North Korea as a democracy, even though this state is officially called “The Democratic People’s Republic of Korea”. Similarly, what is presented as a description of a concept may often be rather a proposal to adopt a certain concept. This may well apply to what Rawls says about *justice*: it seems definitely more appropriate to interpret his definition as a proposal for how the word “justice” should be used than to read it as a description of how the word is in fact used. It clearly violates the principle of charity to point out that justice is traditionally defined as “to render to each their due” and charge Rawls’s description of justice with simply missing the target (as Flew (1985) and Matson (1978) did). Surely, Rawls was not giving a failed attempt at describing the traditional concept of justice but rather insisting on using another concept of justice.

However, the concept-conception distinction does not account for this normative aspect of concept formation. If we try to include it and allow that concepts can be something which a theorist proposes to adopt as a result of theory development, problems arise for the concept-conception analysis of disputes that revolve around contested concepts. After all, one can reasonably surmise that a concept proposed by a defender of a certain conception will not be acceptable as a common ‘core’ to defenders of rival conceptions. They may rather make a rival proposal and argue that we should use another concept (see, e.g. the proposed concepts of freedom discussed in Swanton 1992 or, again, Flew’s (1985) and Matson’s (1978) response to Rawls). In such a situation, defenders of rival concepts and conceptions can still debate about which concept to adopt without talking past each other, but why this is so resists an explanation by the concept-conception approach, because in such cases there is no common concept of, for example, freedom or justice available that explains why the disputants do not talk past each other. In short, the explanation suggested by the concept-conception approach is not viable in cases in which there is a competition between alternative suggestions for concepts.

Moreover, taking the normative aspect seriously has consequences for what counts as an adequate characterization of a concept. In contrast to what the concept-conception theorist holds, the question is not simply whether the actual usage of a term is captured adequately, but at least as much whether a theorist suggests a usage that is in line with the objective she pursues by developing a conception. If we interpret Rawls, for example, as suggesting – as a result of having developed his conception of justice – that we should understand “justice” as making no arbitrary distinctions etc. (Rawls 1999:5, quoted in Sect. 2.1), then the question of whether this is an adequate suggestion cannot be answered by arguments that refer exclusively to the way “justice” is used in the English language. It is also vital to ask whether Rawls’s proposal is adequate in relation to the objective he is pursuing with his conception of justice, roughly an ideal-theory account of justice applicable to the basic structure of society. Other theorists of justice may have different objectives and if so, different suggestions for concepts may count as adequate in relation to their projects and this means that there need not be a single concept providing the common ‘core’. Nonetheless, all disagreeing theorists can acknowledge this and have a dispute without talking past each other – a possibility which cannot be explained with reference to a concept as a common ‘core’ since there is none.^15^

The limitations and challenges discussed in this section are not meant to refute the concept-conception approach to contested concepts. But I take it that they show that Rawls’s idea faces challenges which make it highly questionable whether the concept-conception analysis can successfully be applied beyond exceptionally simple cases such as the debate about enfranchisement. Moreover, that the concept-conception approach resists integrating the dynamic and normative aspects of concept formation indicates that the concept-conception approach cannot do justice to significant aspects of theory development. The remainder of this paper explores how theory development that involves contested concepts can be dealt with by another methodological idea favoured by Rawls, namely, reflective equilibrium. In particular, I want to show that by giving centre stage to the dynamic and normative aspects of concept formation and theory development, reflective equilibrium can better honour the promises which make the concept-conception distinction attractive.

## Reflective equilibrium and conceptual re-engineering

This section introduces the account of reflective equilibrium I rely on. Whereas Section 3.1 adopts the usual perspective, which takes reflective equilibrium to be about epistemic justification, 3.2 explains how reflective equilibrium is also an approach to conceptual re-engineering. This perspective is the basis for investigating (in Sects. 4 and 5) how the reflective-equilibrium approach can be related to the concept-conception distinction and how it can be applied to some of the problems raised by contested concepts.

### A sketch of reflective equilibrium

Goodman (1983) presented his classic account of reflective equilibrium as an answer to the question of how theories of logical validity as well as somebody’s commitments to the logical validity of inferences can be justified. Rawls (1999) then applied reflective equilibrium in the context of theories of justice. Subsequently these ideas have been further developed by a number of authors (e.g. Daniels 1979; DePaul 2011; Elgin 1996; 2017; Scheffler 1954; Tersman 1993) and many varieties have been suggested.^16^ The basic idea of reflective equilibrium is that we start with our commitments about some subject matter, try to find a theory that accounts for the commitments, and then – because in all likelihood there will be conflicts between commitments and the proposed theory – proceed by mutually adjusting commitments and elements of the theory until they agree. The contrast between commitments and theories is not meant to capture a difference in form or content, but to distinguish between, on the one hand, what an epistemic agent believes, accepts, assumes as a working hypothesis or is committed to in some other epistemically relevant way and, on the other hand, what the agent uses for giving a systematic account of the subject matter at hand.^17^ In this way, Goodman, for example, contrasts the inferences a person draws or judges as valid with a logical theory (e.g. *Principia Mathematica*) that provides rules of valid inference; similarly, Rawls mentions “religious intolerance and racial discrimination are unjust” (1999:17) as an example of a commitment and develops a theory of justice based on his two principles of justice and his two priority rules (1999:§ 46).

In the present context, two points are important to note. First, the reflective-equilibrium process has just been described as undertaken by one epistemic agent. This is meant to cover an individual or a group. If the agent is a group, problems related to judgement aggregation or consensus finding, for example, arise ‘within’ the agent. In this paper, however, I do not want to address these issues but the question of what to make of a situation in which different agents develop different positions in reflective equilibrium.

Second, reflective equilibrium has both a static and a dynamic aspect. The idea is that a position in equilibrium, i.e. a state of agreement of commitments and theory, can be reached by a process of mutually adjusting commitments and elements of a theory. Hence we must distinguish between the commitments an agent holds at the initial stage, before the process of equilibrating is started, and her commitments at the resulting stage when an equilibrium is reached. Importantly, the process of mutual adjustments is not intended to give a description of how epistemic agents actually develop their theories, but as a reconstruction of how a resulting position could be reached from some initial commitments, irrespective of whether it has in fact been reached in this way.

The agreement constituting the equilibrium is usually characterized in terms of coherence. At a minimum, this requires that the commitments and the theory are consistent and that the commitments can be inferred (deductively or by some kind of defeasible reasoning) from the theory (and some background assumptions). However, reflective equilibrium is only a plausible account of justification if the target state is not reduced to coherence in the sense just described. To see this, note that so far, since in principle no commitment and no element of a theory is as such immune from revision, nothing rules out that the desired equilibrium could be reached in trivial ways, either by assuming just any theory and adopt whatever can be inferred from this theory as commitments, or by eliminating inconsistencies from initial commitments and declaring the resulting set of commitments to be the theory. Two additional constraints exclude such trivial equilibria.^18^

First, some restrictions are needed to prevent changes to initial commitments which are so drastic that the process of equilibration in fact changes the subject. If, for example, somebody suggested a theory of democracy that rests on the principle that a society is democratic just in case it is lead by a government who decides in the best interest of the people, this would maybe count as an account of beneficial government but not as a theory of democracy, because it would ask us to give up some of our most firm commitments about democracy, for example, that democracy requires that people take in some direct or indirect way part in political decisions. Another example is Rawls who raises the objection of subject-change when he argues that “to each according to his threat advantage is not a principle of justice” (1999:122; see also 116). To prevent such changing of the subject matter, the resulting commitments need to respect the initial commitments in the sense that suitable explanations must be available for revisions of the initial commitments. Such an explanation can point out, for example, that some commitments (e.g. that all and only citizens should have the right to vote) were given up because they were relatively shaky but stood in the way of a highly systematic theory (including, e.g. an all-subjected principle; see Dahl 1989:127).

Second, we must take seriously the idea that we seek not just any old collection of propositions, but a theory which does justice to our epistemic goals. Specifically, we expect a theory to exhibit “theoretical virtues”. For this reason, the literature about reflective equilibrium speaks of *systematic* principles (see, e.g. Daniels 1979; DePaul 2011; Elgin 1996; Scheffler 1954). Theoretical virtues include, to begin with, the general virtues of theories which have been extensively discussed in the literature on theory choice after Kuhn (1977), for example, exactness, broad applicability, simplicity and explanatory power. Specific projects of theory development may also seek to do justice to other virtues such as practical applicability or determinacy in the case of normative theories (Timmons 2013).^19^ Which virtues are relevant and how much weight they should be given depends on the subject matter and the pragmatic-epistemic objective that guides the theory development at hand. In Rawls, for example, this becomes clear when he argues (1999:§ 23) that the objective of his theory of justice (roughly: an ideal-theory account of justice applicable to the basic structure of society) calls for principles that exhibit the virtues of “generality, universality, publicity, ordering, and finality” (1999:126).^20^

### Conceptual re-engineering by reflective equilibrium

Reflective equilibrium is best known and has just been presented as an account of epistemic justification.^21^ But it can, and I think it should, also be understood as an account which simultaneously addresses conceptual engineering in the context of theory development. Before this idea can be discussed in more detail, some general remarks on concepts and conceptual engineering are necessary. Since this is not a paper about the nature of concepts and conceptual engineering, I will confine myself to staking out some basic points with the aim of providing a background that should be acceptable for as many views on concepts and conceptual engineering as possible.

Those who appeal to the concept-conception distinction in discussions that involve contested concepts almost never explicitly rely on a specific theory of concepts. Rawls also does not explain what concepts are. But what he says about the concept-conception distinction rests on the two assumptions that concepts include component notions and have definitions.^22^ In what follows, I want to avoid these assumptions and will use “concept” in a very loose sense as referring to an elementary linguistic entity, a “term”, together with rules for its use.^23^ I take a neutral stance on the nature of such rules. They may determine a term’s extension, intension or some more fine-grained meaning; they may be stated explicitly or remain, in a more or less clear way, implicit in usage; if stated explicitly, they may be given as definitions or in some other form. I also leave open how such rules are related to the mental or abstract entities which are often called “concepts”. By referring to rules for using terms, I adopt a broadly Wittgensteinian approach (see Glock 1996:376–81) as a common framework for discussing terms and their meaning. The basic point I will rely on is that all evidence for a term’s meaning must eventually be found in its use. This leaves open how exactly a term’s meaning can be explained with reference to its use. To do this, one needs to rely on some theory of meaning. In this paper, however, I do not want to presuppose a specific theory of meaning and in particular not the view that meaning can be indentified with use. The way I use “concept” and “meaning” is intended to be compatible with a maximally broad range of theories of meaning and concepts (in some other sense of the word “concept” than the one introduced in this paragraph). In this way, the proposed reflective equilibrium approach to theorizing with contested concepts should be acceptable independent of controversial assumptions about concepts and meaning.

“Conceptual engineering” is used, in this paper, for explicit and intentional concept formation or elimination, and “conceptual re-engineering” for conceptual engineering which is guided by a concept in use and aims at replacing this concept.^24^ Three comments on this. First, speaking of “replacing” one concept by another rather than of “changing”, “modifying” or “transforming” one and the same concept dovetails with the use of “concept” introduced above, which implies that changing the term or the rules for its use yields another concept (see also Carnap 1962:3, 5). This might seem implausible to those who think that many cases of conceptual re-engineering should be described not as replacing but as modifying a concept, for example, if a concept is made more precise. However, if the difference between replacing and modifying is deemed important, one can still introduce criteria that decide under which conditions replacing a concept counts as modifying the original concept rather than as introducing a ‘new’ concept. Since, in what follows, I will not rely on a contrast between replacing and modifying, I will not discuss such criteria. Second, when I subsequently describe theory development as involving conceptual re-engineering, I use this term in a somewhat wider sense insofar as I do not want to imply that those who actually develop the theories understand themselves as engaging in conceptual re-engineering. Third, in contrast to a certain strand in the literature on conceptual engineering, I do not think that conceptual re-engineering needs to be based on a foundational theory of concepts or metasemantics that would justify the claim that conceptual re-engineering is possible at all (e.g. Cappelen 2018). Rather, I take it that theories of concepts need to accommodate the fact that conceptual re-engineering has been successful in a great number of cases. Just think of the many explications in the sciences and in philosophy, ranging from *planet* (IAU 2006) to *self-conscious* (Harris 1990) and from *proposition* (Stalnaker 1984) to *human right* (Griffin 2008).

Moreover, I consider the described assumptions about concepts and conceptual re-engineering to be compatible with the externalist claim that many of the mechanisms involved in determining the meaning of a term are beyond our control (Cappelen 2018). Note that stipulation, as it is customary in, for example science and law, is one of the meaning-determining mechanisms. It can, for instance, hardly be denied that biologists have successfully stipulated which fruits are “berries” for the purposes of plant systematics (see, e.g. Singh 2019:33). The externalist is right in pointing out that this does not by itself change ‘the’ meaning of the English word “berry” at large – laypeople continue to speak of blackberries and strawberries as “berries” – although it can be, as the externalist should admit, a factor that contributes to such a change. All this only shows that some conceptual re-engineering projects are easier and allow for more control (e.g. re-engineering “berry” in plant systematics) than others (re-engineering “berry” at large), but not that there is a puzzle of how conceptual re-engineering is possible at all (see also Koch 2021).

On the basis of these assumptions about concepts and conceptual re-engineering we can now turn to the idea that reflective equilibrium is also an account of conceptual re-engineering in the context of theory development. This idea has virtually gone unnoticed, although it is quite conspicuous in *Fact, Fiction, and Forecast*, where Goodman presents reflective equilibrium as aiming at *defining* “the difference between valid and invalid inductive [and deductive] inferences” (1983:66) and links it to his theory of definition (from Goodman 1977), which is closely related to Carnapian explication.

Explication is a form of conceptual re-engineering which can roughly be described as replacing a concept in use (the “explicandum”) by a concept (the “explicatum”) which is similar to the explicandum but more adequate to some theoretical purpose.^25^ One of Carnap’s examples is the replacement of everyday concepts such as *warm* and *cold* by a concept *temperature* which is exactly defined in physical terms, precisely measurable (on a some scale such as Fahrenheit or Kelvin) and all in all much more fruitful for the purpose of the physical sciences.^26^ Paradigms of philosophical explications can be found, for example, in Hardimon’s (2017) work on *race*, which starts with the customary usage of “race” as the explicandum, analyses a range of theoretical muddles encouraged by this concept and develops four explicata to replace it; for example, a biological concept *populationist race* and a concept *socialrace*, which is geared to the needs of emancipatory critical theory.^27^

The systematic connection between reflective equilibrium and conceptual re-engineering can now be explained as follows: Reflective equilibrium includes an account of conceptual re-engineering as an aspect of theory development insofar as theories determine concepts which serve as explicata for explicanda which, in turn, are characterized by initial commitments.^28^ An example should help to unpack this idea. In the case of *democracy*, we can imagine a theorist who initially holds commitments to propositions such as “Iceland is a democracy”, “democracies have voting systems” or “x is democratic implies that x is not a theocracy”. These commitments settle that the extension of the explicandum “*x* is a democratic state” has Iceland as an element, is contained in the extension of “*x* has a voting system” and that “*x* is a democratic” entails that x is not theocratic. Of course, the theorist will hold a huge number of further commitments, many of them rather trivial (e.g. “democracy is a form of government”) and most of them not explicit (maybe “Vatican was not a democracy in 1974”). A great deal of these commitments will be rooted in established language use, but some may also be the result of previous theoretical efforts and learning by the theorist. Furthermore, the commitments will be more or less firm, their content more or less precise and they may also be in tension or even contradiction with each other – after all, language use can be muddled and inconsistent. Taken together, these initial commitments constitute the theorist’s initial use of the term “democracy”, and provide the evidence for what the rules are the theorist follows in using the term “democracy”. In this sense, the commitments “characterize” the concept of democracy which the theorist employs. To flesh out the details of this relation of characterization is the business of a theory of meaning, which will also need to answer the question of which of the theorist’s commitments are relevant for determining what concepts she employs. For example, an inferentialist (e.g. Peregrin 2014), will hold that a theorist’s commitments to what propositions a statement involving “democracy” entails or is entailed by constitute the evidence for the rules that govern the theorist’s use of “democracy”, whereas others will focus on commitments that show what the theorist is referring to when using “democracy” (see, e.g. Marconi 1997). When the theorist turns to the dynamics of reflective equilibrium, the process of equilibration, she will use her initial commitments as a starting point for developing a political theory. If successful, the result will be a system of concepts and principles which contains definitions or other explicit descriptions of key concepts such as *democracy* or, more specifically, for example *representative* or *parliamentary democracy*, in other words, rules for using “democracy” etc. Given that an equilibrium state requires that the theorist’s commitments agree with the resulting theory, she will also entertain commitments which characterize concepts of democracy, etc. in line with the theory she has developed.^29^ Since for the purpose of theorizing, the resulting concepts replace the concepts characterized by the initial commitments, the reflective-equilibrium process can be interpreted as including a process of explication with the resulting concepts as explicata while the concepts characterized by the initial commitments play the role of explicanda. In this way, theory development and conceptual re-engineering go hand in hand in the process of developing a reflective equilibrium.

Two aspects need further elaboration in the present context. First, at the initial stage of a reflective-equilibrium process, when a theorist sets up a theoretical project, she has to delineate, at least provisionally, the subject matter by selecting relevant commitments. From the perspective of conceptual re-engineering, this includes the first step of an explication, the clarification of the explicandum, as described by Carnap (1962:§ 2). The aim of such a clarification is to eliminate straightforward ambiguity and make a fruitful discussion possible by securing that the usage of the explicandum-term is at least “practically clear”. This requires that the theorist has no qualms about using the term at least in ordinary, simple cases; often the theorist will also be able to give a tentative explanation of the intended meaning of the explicandum-term, which, of course, does not imply that exact rules are given for using the term, let alone a definition. (I’ll come back to the role of practical clarity in Sect. 4.2.) From the reflective-equilibrium perspective, these uses and explanations described by Carnap constitute commitments that go into the initial stage of the reflective-equilibrium process. It remains important to keep in mind that although clarification of the use of terms and delineation of the subject matter are important for setting up a task for theorizing, we must not expect that they deliver concepts that prove to be well suited to the theoretical task at hand. It is plainly realistic to expect that some of our key terms turn out to be ambiguous, their use vague, inconsistent or theoretically unproductive in other ways, and of course, contested. A vivid illustration of these points can be found in a recent book on democracy by Grayling. Right on the first page, we read: “‘Democracy’ has been given many meanings, and the word ‘democratic’ has even been used to describe political systems that are anything but democratic, those typically known as ‘The People’s Democratic Republic of X’. But one system of democracy – *representative democracy* […]” (Grayling 2017:ix). With these statements, Grayling acknowledges the heterogenous uses of “democracy” and makes clear that he intends to use “democracy” in a specific sense as referring to systems of representative democracy and that this is the subject matter he wants to address. Consequently, his commitments pertaining to representative democracy will be relevant to his project, not those pertaining only to direct democracy or some People’s Democratic Republic. Grayling is also quick to point out (2017:x) that a lot of further work is necessary to develop an exact concept of representative democracy, since the meaning of “representative democracy” is contested as well.

Second, it is important to note that although reflective equilibrium has been explained as involving explication – that is, conceptual re-engineering in the service of theory development – this does not mean that reflective equilibrium is restricted to projects which are scientific in some narrow sense. In particular, it leaves room for theoretical activities driven by pragmatic-epistemic objectives that are shaped by social or political aims and values. Reflective equilibrium therefore can be applied, for example, to (at least some of) Haslanger’s well-known so-called ameliorative projects of conceptual re-engineering. When she suggests to give “woman” the sense of (roughly) “[person] subordinated in a society due to their perceived or imagined female reproductive capacities” (Haslanger 2012:8), she intends to provide a concept that proves useful in a critical feminist theory whose objective is to help fight injustice (Haslanger 2000:35–6).^30^ However, including projects shaped by social or political aims and values should not be seen as opening the door to including all kinds of conceptual re-engineering projects. Those which do not involve theory development – for example politically motivated post 9/11 newspeak that re-labels torture as “enhanced interrogation technique” – are not within the scope of reflective equilibrium (nor within the scope of this paper).^31^

## Reflective equilibrium as an alternative to the concept-conception distinction

In order to explore what reflective equilibrium has to offer as an account of theorizing with contested concepts, let us assume that two epistemic agents defend rival theories of, for example democracy, which are justified by reflective equilibrium. In a first step (Sect. 4.1), I analyse whether we can find within the structure of reflective equilibrium some kind of counterpart for the concept-conception distinction. This not only helps to highlight parallels and differences between the two approaches to dealing with contested concepts, but also provides a basis for the second step (Sect. 4.2), which shows how contested concepts can figure in the process of developing a reflective equilibrium and how we can explain why disputes that revolve around contested concepts need not lead into talking past each other.

### Conceptions and concepts in the reflective-equilibrium framework

Since conceptions are theories or parts thereof, they have a straightforward counterpart in the reflective equilibrium framework. They are just (parts of) the theories which have been developed through the mutual adjustments with the commitments.

With respect to the concepts as they figure in the concept-conception distinction, it is less clear whether they have a counterpart in the reflective equilibrium framework. There are in fact two candidates because the dynamic nature of reflective equilibrium introduces a distinction between what we may call “initial” and “resulting” concepts; that is, concepts at the initial and the resulting stage respectively. So we must first look more closely into the difference between initial and resulting concepts.

In the reflective equilibrium framework, initial concepts are characterized by initial commitments. *Justice*, for example, may be initially characterized, inter alia, by commitments concerning paradigmatic cases as, for example, Rawls’s “religious intolerance and racial discrimination are unjust” (1999:17). Although initial concepts result, as we have seen (in Sect. 3.2), from a preliminary step of subject delineation and concept clarification, initial concepts are nonetheless a matter of pre-theoretic^32^ language use and may be inconsistent, underspecified, vague, ambiguous or otherwise ill-suited to the theoretical purpose at hand. They are therefore subject to subsequent revisions effected by adjusting commitments in light of a theory. But even if such revisions are made, initial concepts remain, *qua* initial, unchanged and independent of the resulting theory.

Resulting concepts, on the other hand, are characterized by the resulting commitments in agreement with the theory. They are thus a product of a theory development which most likely involved adjusting commitments. If a state of reflective equilibrium is reached, resulting concepts need to be consistent and at least as theoretically useful as the initial concepts. Consequently, resulting concepts are typically different from initial concepts: they are explicata for the initial concepts. In short, resulting concepts are usually re-engineered concepts.^33^

Now, a problem with the concept-conception approach emerges. It seems that the concepts which figure as the common ‘core’ of rival conceptions must be found at the resulting stage, because this is the stage at which the conceptions will have taken shape and the concepts in question are supposed to be part of the conceptions (as noted in Sect. 2). However, resulting concepts cannot be what corresponds to the concepts to which the concept-conception distinction refers. The reason is that concepts which are shaped by a theory development are exactly not what concepts are supposed to be according to the concept-conception distinction. If, for example, Rawls’s theory characterizes justice with the help of his “first” and “second principle” of justice (Rawls 1999:§ 46), the resulting concept represents what justice is *according to Rawls’s theory* and not what most if not all defenders of rival theories, for example, utilitarians, would accept as the concept of justice.

If we want to look, within the reflective equilibrium framework, for a re-interpretation of the idea of concepts as common ‘cores’, it therefore seems more promising to turn to the initial stage. What we find is that a set of shared initial commitments can provide a common, revisable, starting point for the development of different theories. But such a starting point in shared commitments is in important respects different from a concept as it is understood in the concept-conception approach, which holds that concepts include component notions and have definitions. Although possible, it is by no means guaranteed that the shared initial commitments characterize a concept in this sense. It may also be that theorists disagree about some of the relevant commitments and consequently the shared commitments do not suffice to characterize a common concept, but only ensure that the different concepts employed by the disagreeing theorists share certain features. Two theorists of justice may agree on claims like Rawls’s “religious intolerance and racial discrimination are unjust” (1999:17), while disagreeing on whether “justice” can be described as making no arbitrary distinctions etc. as suggested by Rawls (Rawls 1999:5, quoted in Sect. 2.1). Besides, even if the shared initial commitments characterize a common concept in the sense of the concept-conception distinction, this may not be the common ‘core’ envisaged by the concept-conception approach since there is no guarantee that this concept remains untouched by the process of adjustments and hence it may not be part of the resulting conceptions, as required by the concept-conception approach. All in all, the result so far is that neither initial nor resulting concepts play the role concepts are supposed to play according to Rawls’s concept-conception distinction. This is not to deny that there might be cases in which two theorist share the target concept (and even a definition thereof) at the initial as well as at the resulting stage (*enfranchisement* is a candidate; see Sect. 2.1). But in general, this will not be so, as the considerations given in this section have shown. And this undermines the basis for adopting, within a reflective equilibrium setting, the concept-conception explanation of how we can defend rival theories in which contested concepts play a key role without talking past each other.

### From a common ‘core’ to a shared staring point

An alternative view underpinned by reflective equilibrium is that a sufficient agreement in initial commitments – but not necessarily a common concept – is necessary if a disagreement between defenders of rival theories should not boil down to talking past each other.^34^ Of course, this raises the question of what counts as sufficient agreement in initial commitments. One way to deal with this issue would be to specify a certain kind of commitment two theorists need to share if they should not talk past each other. In this vein, one might claim that the commitments which need to be shared are the commitments that concern language use (“In English, ‘democracy’ is used to refer to a form of government.”) or the commitments which concern conceptual truths or platitudes (“Democracy is a form of government”) rather than merely factual issues (“South Africa is a democracy, but Saudi Arabia is not”). Although such ideas have some plausibility, things are more complicated even if we set aside worries about the nature of platitudes and distinguishing conceptual from factual claims.^35^ One problem is that some commitments about language use seem to be irrelevant (“Nowadays ‘democracy’ is much more frequently used than 200 years ago.”), another is that commitments about paradigmatic instances and non-instances may be as relevant as commitments concerning general conceptual matters or platitudes. All in all, I see little reason to think that a specific kind of commitment could be singled out such that an agreement in initial commitments of this kind is exactly what is necessary to ensure that opponents do not talk past each other.

A more promising alternative is to adapt Carnap’s proposal for clarifying an explicandum (mentioned in Sect. 3.2). The idea is to spell out in terms of practical clarity under what condition an agreement in initial commitments counts as sufficient for not talking past each other: two theorists do not talk past each other in using a term *t* if it is practically clear to them that they use *t* in the same way in the relevant contexts; it is practically clear to *Y* how *X* uses *t* in some context if *Y* is able to predict correctly *X*’s use of *t* for most of the simple, ordinary cases of using *t* in this context (Carnap 1962:4). As Carnap explains, an important means to achieve practical clarity are examples of intended and non-intended uses of the term in focus, but also informal explanations in general terms. In other words, Carnap suggests that the theorists check whether they share commitments about paradigmatic instances and paradigmatic non-instances or commitments that concern general features of the term’s use, which leaves room for commitments of all the kinds discussed in the preceding paragraph.

A number of features of this condition for not talking past each other call for further explanation. First, the condition asks for less than sharing the concept in question since it requires agreement only for most of the simple, ordinary cases. This means that commitments about paradigmatic instances and non-instances play a key role, but an agreement in all cases is not required. Second, the appeal to clarity is practical in the sense of not asking for a theoretically respectable account of the meaning of the term in question, but for a suitable basis for action, namely developing a theory (Carnap 1962:5). This is appropriate for a condition that is meant to be applicable at initial stage, when a theoretically respectable account of the meaning of the term in question is something that still needs to be developed. Third, the condition refers to relevant contexts, because often only a subset of the contexts in which a term is used is relevant to the development of a theory in which this term plays a key role. Hence there is no need to require that two theorists agree across the board in how they use a term. Theorists of *democracy*, for example, may readily agree that they are not interested in uses of democracy which do not refer to a form of government, nor in uses related to some People’s Democratic Republic. Fourth, one might wonder how the suggested condition relates to the idea that two theorists do not talk past each other just in case they both use the target term to talk about the same thing. This raises the question of what one takes to be required for talking about the same thing. If it requires sharing a concept or using a term with exactly the same meaning, then the suggested condition is considerably weaker and does not ensure talking about the same thing in this sense. The main reason for settling on a weaker condition is that we must not assume that at initial stage of inquiry there are neat, exact and consistent rules for using terms such as *democracy, freedom, justice, race* and so on. Rather, an important motivation for developing a theory of, for example, justice is just the observation that the ordinary, ‘non-ideal’, use of “justice” is messy, theoretically unhelpful and poorly understood. But if this is so, requiring that two theorists share a concept or use a term with exactly the same meaning at initial stage would be an unreasonably strong condition for not talking past each other since whether two theorists talk past each other should not depend on whether they agree in unclear or even paradoxical cases. Finally, besides the cases which are the focus of this paper, there are, of course, cases in which applying the suggested condition shows that the two theorists do not use the target term in the same way in the relevant context; in other words, applying the condition may uncover an ambiguity.

The idea that a sufficient agreement in initial commitments can explain why defenders of rival theories need not talk past each other, enables reflective equilibrium to accommodate an attractive aspect of the concept-conception approach. We can retain the idea that, in disputes revolving around contested concepts, defenders of rival theories need not talk past each other as long as they share a suitable point of reference. The crucial differences between the concept-conception approach and the reflective-equilibrium approach concern *what* needs to be shared – commitments rather than concepts – and *when* it needs to be shared – at initial rather than at resulting stage. Whereas the concept-conception approach takes the perspective of the resulting stage and postulates that different conceptions need to share a concept, the reflective-equilibrium approach holds that different reflective-equilibrium processes need to share initial commitments. So instead of the common ‘core’ we have a shared starting point and instead of a shared concept shared commitments.

The shift from a common ‘core’ to a shared starting point is made possible by the dynamic character of reflective equilibrium, which distinguishes stages of theory development. This makes it possible to locate what is shared by disagreeing theorists at the initial rather than at the resulting stage. But a shared starting point in initial commitments is no guarantee for shared concepts at the resulting stage. A sufficient agreement in initial commitments neither implies nor excludes shared resulting concepts since the relevant commitments may be adjusted in different ways by defenders of rival theories. Although it remains possible that, at the resulting stage, the commitments which are part of two different positions in equilibrium characterize a shared concept, this is by no means guaranteed; more likely, there will not be a common ‘core’ in this sense at the resulting stage. This leaves room for the resulting theories to disagree even over the basic structure of a contested concept (see, again, Swanton (1992:7) on *freedom*).

The second shift, from a concept to commitments, has the effect that the turn to reflective equilibrium does not merely result in a more dynamic variant of the concept-conception approach, but also in a much more flexible framework. Defenders of rival accounts of, for example, democracy need not start out with a set of shared commitments that actually characterizes an initial concept of democracy. Instead they may share a diverse range of commitments, large numbers of them utterly uncontroversial. They are likely to include commitments concerning conceptual features of *democracy* (e.g. “Democracy is a system of government in which people rule”) or the relation of *democracy* to other concepts (“*Democracy* and *theocracy* are contraries.”), commitments about language use (“‘Democracy’ refers to a form of government”) but also many commitments about particular (non)instances (“Ancient Athens was democratic, Sweden is, but North Korea is not.”) and general claims about democracy (“Democracy requires voting.”). Furthermore, commitments can differ in weight, ranging from very strong to merely accepted as a working hypothesis (“South Africa is a democracy now.” vs. “South Africa has been a democracy since 1994.”). Differences between theorists at the initial stage therefore need not invariably pertain to the commitment’s content but may also consist in different weighing. Maybe one theorist is less willing than another to give up the commitment that ancient Athens was a democracy. All this makes, I think, also for an account that is much more realistic than the assumption that defenders of rival theories share a concept.

However, one might worry whether the idea described in this section is really viable. A problem seems to arise, when we want to assess whether two theorists share some initial commitment. If, for example, we want to say that two theorists share the commitment “Macedonia is democratic” and that this shows that their use of “democratic” agrees in the case of Macedonia, we surely need to assume that they agree in their use of “Macedonia”. If one theorist referred to the state now officially named “North Macedonia” but the other to the Greek region named “Macedonia”, then the fact that they both assent to “Macedonia is democratic” would not show that they agree about an actual instance of a democratic governmental body. They would rather talk past each other with respect to “Macedonia”. Whether this is so is, on the account proposed here, a question of whether they sufficiently share commitments related to Macedonia. So it seems we are off on a regress of commitments that need to be shared.

How troublesome is this worry? First, we should note that it is not a challenge specifically for the reflective-equilibrium approach. It affects the concept-conception approach even more. This approach relies on the assumption that the disputing theorists share a concept which is defined in terms of some component notions. But such a definition only secures a common concept if the theorists share these component notions (as mentioned in Sect. 2). At this point, defenders of the concept-conception approach have two options. They can treat the component notions as concepts with their own definitions in terms of further component notions, which means going into the next round of a definitional regress. Alternatively, they can avoid the regress by defending the assumption that we share the component notions in some other way. In many specific cases, assuming shared component notions seems to be warranted as long as there is no indication to the contrary: if our disagreement focuses on *democracy*, we may safely assume that we use “Macedonia” in the same way, because if we do not, this seems easy enough to detect. Of course, the same point can be made in defence of the reflective-equilibrium approach. But plausibility in particular cases aside, defenders of the concept-conception approach owe us a story of how we can make sure that the disputing theorists actually share the component notions without resorting to definitions. And this challenge is harder to meet for the concept-conception approach than the corresponding challenge for the reflective-equilibrium approach. After all, the reference to a shared staring point in initial commitments was introduced as a less demanding alternative to a common concept.

More is required, however, to fully answer the objection. From a reflective-equilibrium perspective the best move, I think, is to admit that the requirement of a sufficient agreement in initial commitments starts a cascade of further requirements to share other commitments as well. For example, sufficient agreement in initial commitments about Macedonia is needed if an agreement with respect to “Macedonia is democratic” should contribute to securing a shared starting point for rival theories of democracy. This move is motivated by the reflective equilibrium approach for independent reasons. Since reflective-equilibrium was introduced as an account of theory development, it was clear from the beginning, that a reflective-equilibrium process has to start with a relatively broad range of commitments pertaining to some subject matter, rather than with commitments related to just some isolated concept. Furthermore, subject matters cannot be dealt with completely independently. As Elgin (2017:ch. 4; see also Baumberger/Brun 2021) and Tersman (1993:ch 5.3) have argued, a tendency towards holism is therefore built into reflective-equilibrium, even if in practice, theory development must proceed piecemeal.

## Theoretical disagreements involving contested concepts

The dynamic perspective of an equilibration process and the two shifts described in the last section provide the basis for shedding new light on theoretical disagreements involving contested concepts. In a nutshell, the suggested picture is this: if different positions in reflective equilibrium are developed from a starting point in which there is sufficient agreement in initial commitments, disagreement results; since the disagreement concerns the resulting theories and consequently also the resulting commitments and the concepts they characterize, we end up with contested concepts; but the shared starting point can explain why the resulting disagreement need not lead to talking past each other. Let us develop this perspective in more detail and see what it can contribute to understanding theorizing in which contested concepts plays a key role.

To begin with, the possibility that different theorists can defend different theories without thereby talking past each other can be explained with reference to a shared starting point in initial commitments that makes practically clear that the theorists sufficiently agree in their pre-theoretical use of the target term (as discussed in the preceding section): if the rival theorists have identified such a starting point, they have sufficient reason to believe that they set out to understand at least roughly the same thing, even if at the resulting stage they defend different concepts and maybe rely on alternative uses of the same term – in which case it is also inappropriate to describe the resulting situation as involving one and the same contested concept. In short, an agreement at the initial stage explains why a disagreement at the resulting stage is possible without talking past each other.

However, it should also be noted that even if there is such a shared starting point, it remains easy to talk past each other. The perspective developed in this paper can help to understand better some of the ways in which this can happen. One type of potentially problematic situation are cases in which the two theorists simply assume, albeit erroneously, that they sufficiently agree in initial commitments. If they both use the same pre-theoretical language, such an assumption may easily seem justified although an attempt to make practically clear that they sufficiently agree in their use of the target term would reveal that the assumption is unwarranted. As Carnap remarks (1962:4), frustratingly often no careful attempt at reaching the required practical clarity is made. Another source of talking past each other are situations in which two theorists have ended up adopting two markedly different re-engineered concepts, but fail to be duly aware of this. The danger of talking past each other for this reason increases vastly if resulting concepts are not properly distinguished from initial concepts, which, in turn, is greatly facilitated by using the same term for both the initial and the resulting concepts. Without these distinctions, intractable controversies can result, because it will remain unclear whether there is a shared starting point that can be used to explain the rival theories as the result of taking, from there, diverging routes of theory development.

Second, the reflective-equilibrium approach can explain the normative aspect of controversies over concepts. In Section 2, it was observed that theorists often suggest that some terms should be used in certain ways rather than others; that is, they suggest that certain concepts should be adopted. Rawls and Haslanger, for example, suggest that we should adopt specific concepts of justice and woman respectively. They do so by giving definitions for “justice” and “woman” which introduce an explicatum for the pre-theoretical concepts *justice* and *woman*. What is the basis for such normative claims that a specific explicatum should be adopted? From a reflective-equilibrium perspective, the general answer is that the suggested explicata should be adopted because they are part of a theory which is epistemically justified by reflective equilibrium in relation to the pragmatic-epistemic objective which drove the development of the theory. This is so because the reasons which speak in favour of adopting a position in reflective equilibrium are also reasons to employ certain concepts rather than others, namely the resulting concepts rather than the initial concepts. Since these reasons are sensitive to the pragmatic-epistemic objectives which guide the development of the theory at hand, the normative force of suggestions that the resulting explicata should be adopted is primarily epistemic, but has also a pragmatic aspect, the exact nature of which depends on the objective guiding the theory development.

For example, the reasons why the concept of justice described by Rawls – justice as making no arbitrary distinctions etc. (see the quote at the beginning of Sect. 2.1) – should be adopted are to be sought in what justifies his theory of justice. Showing that Rawls’s theory of justice is justified by reflective equilibrium requires to argue, for instance, that adopting his concept of justice permits one to respect one’s initial commitments about justice (e.g. “religious intolerance and racial discrimination are unjust”; Rawls 1999:17) and that adopting his concept is part and parcel of adopting principles which exhibit the theoretical virtues (“generality, universality, publicity, ordering, and finality”; 1999:126) Rawls holds to be required by his objective of formulating (roughly) an ideal-theory account of justice applicable to the basic structure of society (1999:6–9). In case of Haslanger’s concept *woman*, the situation is somewhat different insofar as her re-engineering proposal for *woman* (roughly: “person who is systematically subordinated in virtue of being perceived as having female reproductive capacities”; Haslanger 2000:42–3) is not embedded in a specific project of theory development, but merely claimed to be useful in critical feminist theories that aim at fighting injustice (2000:35–6). From a reflective-equilibrium perspective, however, the question of whether the claim that her concept *woman* should be adopted must be assessed in the context of a specific theory. Assuming we had a critical feminist theory that included Haslanger’s definition and that this theory was in reflective equilibrium, the reasons for adopting the re-engineered concept *woman* would be epistemic and instrumental in relation to the practical aim of fighting injustice.

Third, there are the questions of how it is possible to reach rival theories and of how we should interpret the resulting disagreement. A full discussion of the epistemology of disagreement from a reflective-equilibrium perspective cannot be undertaken in this paper, but here is a key observation: two theorists can begin from a starting point of a set of shared initial commitments or even from the same set of initial commitments but reach different reflective-equilibrium states with different theories that characterize key concepts differently – as a result, these concepts can be contested. Such divergent courses of theory development are possible for a number of reasons. Some of them are not particular to reflective-equilibrium and are also discussed in the literature on conceptual engineering (see, e.g. Kitcher 2008; Olsson 2017). For example, different background assumptions may be responsible for different theorists reaching different theories, and the resulting disagreement just reflects those differences in background assumptions. Another possibility is that different theories are tailored to different pragmatic-epistemic objectives, and since this, in turn, can affect how the epistemic virtues are interpreted and weighed against each other different positions may count as being in reflective equilibrium. If different theories of the same subject matter can in this way be traced to differences in background assumptions or pragmatic-epistemic objectives, the resulting disagreement can be explained away as ‘harmless’ insofar as it is only a secondary result of differences in background assumptions or pragmatic-epistemic objectives. Nonetheless, such cases of different theories can lead to talking past each other if it remains unnoticed why the different theories have been developed. If this is the case, a concept may be contested because it goes unnoticed that two theorists rely on different background assumptions or pursue different pragmatic-epistemic objectives and therefore defend different theories and consequently also different concepts.

However, reflective equilibrium has also room for other ways of arriving at alternative theories which give rise to genuine pluralism (as Goodman emphasized, e.g. in 1983:63; see also Elgin 1996: 134–45). Two theorists can start from the same ‘initial conditions’ and still reach different positions in reflective equilibrium. This can happen if they share initial commitments, pursue the same pragmatic-epistemic objective, rely on the same background assumptions, but go through different processes of adjustments. Reflective equilibrium allows this because the process of theory development is not deterministic and may permit different, equally defensible decisions in three respects. For one thing, the process is path dependent. Different theorists may choose to address mismatches between theory and commitments in a different order, and consequently arrive at different positions. For another, the considerations which guide the choice of the necessary adjustments of the theory or the commitments will often not determine a unique outcome. Usually, we have to deal with considerable indeterminacy when we have to assess the relative weight of commitments and theoretical virtues, what trade-offs are admissible, or how exactly a certain theoretical virtue may be interpreted. How, for instance, do we understand simplicity in a given context? How much of it might be traded off against gains in practical applicability or scope of application? Instead of relying on some formal procedure, the theorist will need to make a judgement on the relative weight of the different epistemic virtues and of the relative force of the two antagonistic requirements of doing justice to epistemic virtues and respecting initial commitments.^36^ Finally, the choice between the different adjustments on offer may be underdetermined, because several overall equally acceptable adjustments can be available.^37^ In such cases, a theorist may need to proceed by picking an option.

Whenever two theorists reach different states of reflective equilibrium by taking different decisions in one of the three described ways, pluralism results in so far as one theorist must admit that the other’s position is as justified as her own, because she can see that both positions are the result of a reflective-equilibrium process that started from the same initial position. If this happens, the result is a disagreement which is not based on talking past each other and this situation raises a host of epistemic questions known from the debate about peer disagreement (see, e.g. Feldman/Warfield 2010; Christensen/Lackey 2013), for example, whether defenders of rival theories should actually suspend commitment, whether they are rationally permitted to remain steadfast or in which ways they rationally required to modify their position. Discussing such questions with reference to rival reflective equilibria must be left to a paper of its own.

## Conclusion and outlook

This paper has outlined a reflective-equilibrium based approach to some of the problems raised by theoretical debates in which contested concepts play a key role. From the reflective-equilibrium perspective, it becomes clear that and how theory development involves not only articulation but re-engineering of contested concepts. This approach, I have argued, is more promising than the popular idea of relying on the concept-conception distinction, because reflective equilibrium can incorporate attractive features of the concept-conception distinction, but better account for the normative and dynamic aspects of debates that revolve around contested concepts. In particular, we can give a more flexible and realistic explanation of how it is possible to have disputes driven by contested concepts without talking past each other. Moreover, the reflective-equilibrium approach provides an understanding of various ways in which contested concepts can lead to disagreements in such disputes. The decisive shift is to think of disagreements driven by contested concepts not in terms of rival conceptions tied to each other by a common ‘core’ of a shared concept, but in terms of rival theoretical positions reached from a shared starting point in initial commitments couched in pre-theoretic language use. In this way, the reflective-equilibrium approach promises to provide a basis for making progress in the epistemology of disagreements driven by contested concepts.

Finally, I would like to briefly remark on the scope of application of the proposed analysis. Contested concepts are, of course, to be found not only in social and political philosophy, but in all areas of philosophy and in many other fields of research. If the proposed analysis is convincing, it is available for all contexts in which one is ready to defend reflective equilibrium. If one thinks that reflective equilibrium is an account of what epistemic justification amounts to in philosophy (e.g. Lewis 1983:x, Keefe 2000:ch. 2) or in general (e.g. Elgin 2017), we should be able to apply the reasoning proposed in this paper to debates about concepts such as *pain* (Reuter/Sytsma 2020), *happiness* (Martin 2008) or *person* (Rawls 1980:571). Furthermore, nothing in the proposed reflective-equilibrium approach really depends on whether it involves concepts that are contested. This does not only mean that the approach can be applied more generally, but also that it shows how debates involving contested concepts are the result of general processes of theory development and conceptual re-engineering.

## Footnotes

1 I follow the convention of using italics for referring to concepts.

2 Following Gallie 1956, it is sometimes held that certain concepts are not only contested but *essentially* contested. In this paper, I do not rely on this idea and do not aim at analysing or defending it.

3 Two exceptions are Swanton 1992 and Criley 2007, which both take an approach that differs considerably from the one I take in this paper. Firstly, the account of reflective equilibrium I introduce is more elaborate and in many respects distinct from both Swanton’s and Criley’s. Secondly, conceptual engineering plays a key role in this paper, but it is considered neither by Swanton nor by Criley. Finally, Criley builds the concept-conception distinction into the very definition of “contested concept” (2007:ch. 1.3), whereas this paper explores reflective equilibrium as an alternative to the concept-conception distinction.

4 This is also true of the other methodological ideas discussed in this paper, namely explication and reflective equilibrium, which were canonically described in Carnap 1956, 1962, 1963, Goodman 1983 and Rawls 1999 respectively. Rawls’s description of reflective equilibrium particularly well illustrates the problematic status of such methodological remarks since his presentation amalgamates reflective equilibrium with aspects of his contractualism in a way that makes it quite unclear what he took to be reflective equilibrium *in general*.

5 Rawls (1999:5) attributes the idea of distinguishing concepts and conceptions to Hart (1994).

6 Applications include: *autonomy* (Seidel 2016:ch. 1.4–5), *democracy* (Connolly 1993:10–11; List/Valentini 2016:532), *happiness* (Martin 2008:175), *justice* (Rawls 1999:5), *person* (Rawls 1980:571), *politics* (Connolly 1993:ch. 1), *power* (Lukes 2005:30), *race* (Hardimon 2017), *sustainable development* (Jacobs 1999).

7 Suggestions for distinguishing varieties of the concept-conception distinction can be found, e.g. in Lalumera 2014 and Swanton 1992:2–3.

8 I will therefore not investigate what role the concept-conception distinction actually plays in Rawls’s work, nor will I attempt to ‘reverse engineer’ a methodology from how Rawls actually proceeds in his work on justice.

9 As an example of straightforward context-dependency we can think of “unusual punishment” which refers to different practices at different times and in different communities (Lanius 2019:25).

10 I will continue using the expression “contested concepts” when I refer to the problems this paper discusses, although from a Rawlsian perspective this is a misnomer since the ‘real’ debate is about conceptions, not concepts.

11 The plausibility of this example is partially due to the fact that it seems at least prima facie possible to specify the meaning of “enfranchised” (as having voting rights) independently of deciding for any specific group (residents, children, etc.) whether its members are in its extension (have a voting right) and why. This might be disputed, e.g. by claiming that different competence requirements are linked to different notions of voting. For the sake of argument, I ignore this objection and will not discuss whether it could be turned into a general critique of Rawls’s explanation of the concept-conception contrast although one might think that a neat separation of specifying meaning and specifying extension is not available for the standard cases discussed in the literature, including Rawls’s *justice*.

12 I use “*x* is a hyperonym of *y*” for the converse of the asymmetrical relation “*x* is a hyponym of *y*”; so *flower* is a hyperonym of *rose*, *violet*, *daffodil*, etc., which are all hyponyms of *flower* (see Lyons 1977:291).

13 Since analogous problems arise if the common ‘core’ is the meaning of a polysemous term with a range of different senses, I will not rely on a distinction between ambiguity and polysemy in what follows.

14 An exception is D’Agostino’s (1996:ch. 9–15) analysis of conceptions of public justification, which, however, is un-Rawlsian since he holds that the concept of public justification is “multiply ambiguous” (1996:30).

15 The normative aspect of concept formation will be picked up again in Sect. 5, where I briefly discuss it from the perspective of reflective equilibrium.

16 A detailed exposition of the account of reflective equilibrium sketched in this section and more examples can be found in Baumberger/Brun 2016, 2021, Brun 2014, 2020, which in turn draw on Elgin’s work. For extended case-studies on the application of RE, see Rechnitzer forthcoming. In this paper, I simplify in two important respects by largely ignoring, firstly, the role background theories play in so-called wide reflective equilibrium and, secondly, the requirement that some of the resulting commitments have independent credibility.

17 The use of “commitment” in this technical sense goes back to Scheffler 1954. However, the literature very often does not contrast commitments with theory, but rather speaks of “judgements” and “principles” and characterizes their contrast as one of particular vs. general. This is problematic as a general explanation of reflective equilibrium because, inter alia, it excludes general judgements – leading to the bizarre effect that general statements such as “religious intolerance and racial discrimination are unjust” (Rawls 1999:17), “slavery is unjust” and “income inequity is unjust” (Criley 2007:139) are given as alleged examples of particular judgements (see Brun 2014 and 2020 for further discussion).

18 There is a further constraint which requires independent credibility of some resulting commitments. Since it plays no important role in the present context, I will not discuss it. As an additional simplification, I will also ignore the fact that commitments can emerge between the initial and the resulting stage. For a discussion of these points, see the references in note 16.

19 See Douglas 2013 for a recent overview of virtues discussed in general philosophy of science, and Rechnitzer 202*:ch. 5.5 for virtues of ethical theories.

20 Detailed examples of how pragmatic-epistemic objectives guide the choice and weighing of theoretical virtues in the sciences can be found in, e.g. Elliott/McKaughan 2014.

21 For present purposes, it can be left open what the exact nature of the justification provided by reflective equilibrium is (e.g. whether it is truth conducive). Some authors, most prominently DePaul (e.g. 2011), see reflective equilibrium as an account of some epistemically relevant status they take to be different from justification.

22 One might therefore wonder whether Rawls’s concept-conception distinction presupposes the so-called classical theory of concepts and more generally whether it is open to some of the objections against the classical theory (see, e.g. Laurence/Margolis 1999). I will not pursue this issue in this paper.

23 When I refer to concepts in the sense of the concept-conception distinction in the rest of this paper, I will flag this explicitly if the context leaves room for doubt.

24 Clearly, theory development often includes forms of conceptual engineering which are not cases of *re-*engineering since they do not replace a concept but eliminate a concept or introduce a new concept that is not supposed to replace a pre-existing concept. Insofar as such forms of conceptual engineering can lead to disputes whether some concept should be eliminated or introduced, one could speak of “contested concepts” in such cases as well. This, however, is not what the debate about contested concepts focuses on and I will therefore not discuss such forms of conceptual engineering in this paper.

25 The classic exposition of explication is Carnap 1962:ch. I; see Brun 2016 for a detailed discussion.

26 See Carnap 1962:§§ 4–5. Chang’s (2004) seminal analysis of the actual development of the concept *temperature* as a process of “epistemic iteration” shows that its long and involved story fits well into the paradigm of reflective equilibrium.

27 Here is Hardimon’s definition of *populationist race*: “a subdivision of *Homo sapiens* – a group of populations that exhibits a distinctive pattern of genetically transmitted phenotypic characters that corresponds to the group’s geographical ancestry and belongs to a biological line of descent initiated by a geographically separated and reproductively isolated founding population” (Hardimon 2017:99).

28 The interpretation of reflective equilibrium as an account of conceptual re-engineering as well as the systematic and historical links between Goodman’s and Carnap’s methodological work are discussed in Brun 2020.

29 In general, one needs to distinguish between the concepts which are determined by the resulting theory and the concepts characterized by the resulting commitments (see Brun 2020). Since for present purposes, this distinction is not important, I will ignore it for the sake of simplicity.

30 See Mühlebach 2016 for a discussion of the relation between ameliorative projects and reflective equilibrium, and Dutilh Novaes 2020 for the relation between ameliorative projects and explication.

31 It is, of course, not always clear whether a project of conceptual re-engineering aims at theory development. Haslanger, e.g. sometimes describes her project of engineering *woman* as aiming directly at social change, quite independent of any theory development (e.g. 2000:47–8).

32 I use “pre-theoretic” in the sense of independent of the current activities of theory development, not in the sense of prior to any theoretical considerations whatsoever.

33 Note that I do not claim that initial concepts invariably are unsuited for theoretical purposes, nor that resulting concepts must always differ in meaning from the corresponding initial concepts.

34 I do not argue that the account I develop in this paper is the only possible approach to disputes about contested concepts from a reflective-equilibrium perspective, but exploring alternative reflective-equilibrium based approaches lies beyond the scope of this paper.

35 In addition to the well-known arguments against a difference in kind between conceptual and factual statements (e.g. Quine 1951), there are also theories of meaning according to which some apparently factual claims are conceptual. For example, an exemplar theorist of meaning (see Hampton 2016 for an overview) would hold that statements noting paradigmatic instances make conceptual claims.

36 Some of these points are, of course, well-known from debates about theory choice in philosophy of science in the wake of Kuhn 1977; see also note 20.

37 I use “overall equally acceptable” rather than “equally good” in order to leave open that the options cannot be assessed in terms of better/worse/equally good, but only as roughly equal or on a par (see Hsieh 2016).

## References

[CR1] Baumberger C, Grimm SR, Christoph B, Ammon S (2016). “Dimensions of Objectual Understanding”. Explaining Understanding. New Perspectives from Epistemology and Philosophy of Science.

[CR2] Baumberger C (2021). Reflective Equilibrium and Understanding. Synthese.

[CR3] Beckman L (2009). The Frontiers of Democracy. The Right to Vote and its Limits.

[CR4] Brun G (2014). “Reflective Equilibrium without Intuitions?. Ethical Theory and Moral Practice.

[CR5] Brun G (2016). Explication as a Method of Conceptual Re-engineering. Erkenntnis.

[CR6] Brun G (2020). Conceptual Re-Engineering: From Explication to Reflective Equilibrium. Synthese.

[CR7] Cappelen H (2018). Fixing Language. An Essay on Conceptual Engineering.

[CR8] Carnap, R. (1956). [1947]. *Meaning and necessity. A study in semantics and modal logic* (2nd. ed.). Chicago: University of Chicago Press

[CR9] Carnap, R. (1962). [1950]. *Logical foundations of probability* (2nd ed.). Chicago/London: University of Chicago Press/Routledge and Kegan Paul

[CR10] Carnap R, Schilpp P, Arthur (1963). “Replies and Systematic Expositions”. The Philosophy of Rudolf Carnap.

[CR11] Chang H (2004). Inventing Temperature. Measurement and Scientific Progress.

[CR12] Christensen D, Lackey J (2013). The Epistemology of Disagreement. New Essays.

[CR13] Connolly WE (1993). The Terms of Political Discourse.

[CR14] Criley, M. E. (2007). *Contested concepts and competing conceptions.* PhD thesis, University of Pittsburgh. http://d-scholarship.pitt.edu/id/eprint/9832 (30.06.20)

[CR15] D’Agostino F (1996). Free Public Reason. Making It Up As We Go.

[CR16] Dahl RA (1989). Democracy and Its Critics.

[CR17] Daniels N (1979). Wide Reflective Equilibrium and Theory Acceptance in Ethics. The Journal of Philosophy.

[CR18] DePaul MR, Miller C (2011). “Methodological Issues. Reflective Equilibrium”. The Continuum Companion to Ethics.

[CR19] Douglas H (2013). The Value of Cognitive Values. Philosophy of Science.

[CR20] Dutilh Novaes C (2020). Carnapian Explication and Ameliorative Analysis. A Systematic Comparison. Synthese.

[CR21] Elgin CZ (1996). Considered Judgment.

[CR22] Elgin CZ (2017). True Enough.

[CR23] Elliott KC, Daniel JMK (2014). Nonepistemic Values and the Multiple Goals of Science. Philosophy of Science.

[CR24] Feldman R, Warfield TA (2010). Disagreement.

[CR25] Flew A (1985). The Concept, and Conceptions, of Justice. Journal of Applied Philosophy.

[CR26] Gallie, W. B. (1956). Essentially contested concepts. *Proceedings of the Aristotelian Society* new series 56, 167–198

[CR27] Glock HJ (1996). A Wittgenstein Dictionary.

[CR28] Goodman, N. (1977). [1951]. *The structure of appearance* (3rd ed.). Dordrecht/Boston: Reidel

[CR29] Goodman, N. (1983). [1954]. *Fact, fiction, and forecast* (4th ed.). Cambridge, MA: Harvard University Press

[CR30] Grayling AC (2017). Democracy and Its Crisis.

[CR31] Griffin J (2008). On Human Rights.

[CR32] Hampton, J. A. (2016). “Categories, prototypes and exemplars”. In N. Riemer (ed.), *The Routledge handbook of semantics* (pp. 125–141). London/New York: Routledge

[CR33] Hardimon MO (2017). Rethinking Race. The Case for Deflationary Realism.

[CR34] Harris PR, Crozier W, Ray (1990). “Shyness and Embarrassment in Psychological Theory and Ordinary Language”. Shyness and Embarrassment. Perspectives from Social Psychology.

[CR35] Hart, H. L. A. (1994). [1961]. *The concept of law* (2nd. ed.) Oxford: Clarendon Press

[CR36] Haslanger S (2000). Gender and Race. (What) Are They? (What) Do We Want Them To Be?. Noûs.

[CR37] Haslanger S (2012). Resisting Reality. Social Construction and Social Critique.

[CR38] Hsieh, N. (2016). “Incommensurable Values”. In *Stanford Encyclopedia of Philosophy*. https://plato.stanford.edu/archives/spr2016/entries/value-incommensurable/

[CR39] International Astronomical Union [IAU] (2006). *Resolution B5. Definition of a planet in the solar system*, http://www.iau.org/static/resolutions/Resolution_GA26-5-6.pdf (30.06.20)

[CR40] Jacobs M, Dobson A (1999). “Sustainable Development as a Contested Concept”. Fairness and Futurity. Essays on Environmental Sustainability and Social Justice.

[CR41] Keefe R (2000). Theories of Vagueness.

[CR42] Kitcher P (2008). Carnap and the Caterpillar. Philosophical Topics.

[CR43] Koch S (2021). The Externalist Challenge to Conceptual Engineering. Synthese.

[CR44] Kuhn, T. S. (1977). “Objectivity, value judgment, and theory choice”. In *The essential tension. Selected studies in scientific tradition and change* (pp. 320–339). Chicago: University of Chicago Press

[CR45] Lalumera E (2014). On the Explanatory Value of the Concept-Conception Distinction. Rivista Italiana di Filosofia del Linguaggio.

[CR46] Lanius, D. (2019). *Strategic Indeterminacy in the Law*. New York: Oxford University Press

[CR47] Laurence S, Margolis E, Laurence S (1999). “Concepts and Cognitive Science”. Concepts. Core Readings.

[CR48] Lewis D (1983). Philosophical Papers. Vol I.

[CR49] List, C. & Valentini, L. (2016). “The methodology of political theory”. In H. Cappelen, T.S. Gendler, & J. Hawthorne. *The Oxford Handbook of Philosophical Methodology* (525–553). Oxford: Oxford University Press.

[CR50] Lukes S (2005). Power. A Radical View.

[CR51] Lyons J (1977). Semantics.

[CR52] Marconi D (1997). Lexical Competence. Language, Speech, and Communication.

[CR53] Martin MW (2008). Paradoxes of Happiness. Journal of Happiness Studies.

[CR54] Matson W (1978). What Rawls Calls Justice. The Occasional Review.

[CR55] Mühlebach D (2016). Reflective Equilibrium as an Ameliorative Framework for Feminist Epistemology. Hypatia.

[CR56] Newman JA, Gary V (2017). Defending Biodiversity. Environmental Science and Ethics.

[CR57] Olsson EJ, Coliva A, Pedersen NJLL (2017). “Explicationist Epistemology and Epistemic Pluralism”. Epistemic Pluralism.

[CR58] Peregrin J (2014). Inferentialism. Why Rules Matter.

[CR59] Plunkett D (2015). Which Concepts Should We Use? Metalinguistic Negotiations and The Methodology of Philosophy. Inquiry.

[CR60] Plunkett D (2013). Disagreement and the Semantics of Normative and Evaluative Terms. Philosopher’s Imprint.

[CR61] Quine W, Van Orman (1951). Two Dogmas of Empiricism. The Philosophical Review.

[CR62] Rawls J (1980). Kantian Constructivism in Moral Theory. The Journal of Philosophy.

[CR63] Rawls J (1999). A Theory of Justice. Revised edition.

[CR64] Rawls J (2005). Political Liberalism. Expanded ed.

[CR65] Rechnitzer, T. (Forthcoming). *Applying reflective equilibrium. Towards the justification of a precautionary principle*. Springer

[CR66] Reuter K (2020). Unfelt pain. Synthese.

[CR67] Scheffler I (1954). On Justification and Commitment. Journal of Philosophy.

[CR69] Seidel C (2016). Selbst bestimmen. Eine philosophische Untersuchung personaler Autonomie.

[CR70] Singh G (2019). Plant Systematics. An Integrated Approach.

[CR71] Stalnaker, R. (1984). *Inquiry.* Cambridge, MA: MIT Press

[CR72] Swanton C (1992). Freedom. A Coherence Theory.

[CR73] Tersman F (1993). Reflective Equilibrium. An Essay in Moral Epistemology.

[CR74] Timmons M (2013). Moral Theory. An Introduction.

